# Biofilms on glacial surfaces: hotspots for biological activity

**DOI:** 10.1038/npjbiofilms.2016.8

**Published:** 2016-06-08

**Authors:** Heidi J Smith, Amber Schmit, Rachel Foster, Sten Littman, Marcel MM Kuypers, Christine M Foreman

**Affiliations:** 1 Center for Biofilm Engineering, Montana State University, Bozeman, MT, USA; 2 Land Resources and Environmental Sciences, Montana State University, Bozeman, MT, USA; 3 Chemical and Biological Engineering, Montana State University, Bozeman, MT, USA; 4 Department of Ecology, Environment, and Plant Sciences, Stockholm University, Stockholm, Sweden; 5 Department of Biogeochemistry, Max Planck Institute for Marine Microbiology, Bremen, Germany

## Abstract

Glaciers are important constituents in the Earth’s hydrological and carbon cycles, with predicted warming leading to increases in glacial melt and the transport of nutrients to adjacent and downstream aquatic ecosystems. Microbial activity on glacial surfaces has been linked to the biological darkening of cryoconite particles, affecting albedo and increased melt. This phenomenon, however, has only been demonstrated for alpine glaciers and the Greenland Ice Sheet, excluding Antarctica. In this study, we show via confocal laser scanning microscopy that microbial communities on glacial surfaces in Antarctica persist in biofilms. Overall, ~35% of the cryoconite sediment surfaces were covered by biofilm. Nanoscale scale secondary ion mass spectrometry measured significant enrichment of ^13^C and ^15^N above background in both *Bacteroidetes* and filamentous cyanobacteria (i.e., *Oscillatoria*) when incubated in the presence of ^13^C–NaHCO_3_ and ^15^NH_4_. This transfer of newly synthesised organic compounds was dependent on the distance of heterotrophic *Bacteroidetes* from filamentous *Oscillatoria*. We conclude that the spatial organisation within these biofilms promotes efficient transfer and cycling of nutrients. Further, these results support the hypothesis that biofilm formation leads to the accumulation of organic matter on cryoconite minerals, which could influence the surface albedo of glaciers.

Glaciers cover roughly 10% of Earth’s land surface. As the Earth warms, losses in glacial mass will lead to an export of freshwater to marine ecosystems and ultimately a rise in sea level. Not only is the sheer volume of freshwater of concern, but also the release of geochemical constituents in glacial ice. It is well-known that life in icy systems is predominantly microbial, but little is known about the direct role of microorganisms in biogeochemical cycling from these environments. Of particular interest for carbon cycling are glaciological features known as cryoconite holes, which contain both mineralogical and biological materials. Cryoconite holes form as aeolian debris melts into ice surfaces, and may cover a significant proportion of the ablation zone of glaciers (3.2–16%) in the Southern and Northern Hemispheres.^1–3^ These features are hotspots of microbial activity, capable of fixing significant amounts of carbon.^4^ Sophisticated models exist to explain carbon fluxes in cryoconite, but due to a lack of data these models have explicitly excluded Antarctic cryoconite holes.

Although research on the role of biological processes on glacier surfaces is still in its infancy, recent reports have linked biological growth to a reduction in glacial surface albedo through increased surface darkening and enhanced melting.^5^ Biologically driven aggregation of particles is influenced by the quantity, quality and decomposition of organic matter (OM), and strong correlations between granule size and OM content have been reported.^6^ Binding together these aggregates are living matrices, called biofilms, which enhance cell–cell and cell–particle interactions, leading to a positive-feedback increasing biomass and potentially reducing albedo on glacial surfaces. While granules, dominated by cyanobacteria and extracellular polymeric substances (EPS), are evident in Arctic and higher latitude cryoconite ecosystems,^6^ granules in Antarctic cryoconite have not been observed^7^ and information on cell–particle interactions is non-existent. In a recent review, Cook *et al.*^6^ emphasise the need for understanding the fundamental mechanisms that control cryoconite holes from different geographical locations, in order to develop general models that are applicable to diverse supraglacial environments. We propose that the biofilm matrix is integral to biological activity in cryoconite holes through enhanced nutrient storage, cell adhesion and the promotion of efficient nutrient transfer between community members. As such, development of the biofilm structure supports entrapment of microbes and OM, stimulating cell adhesion to cryoconite particles. This accumulation of microbes and OM will enlarge these aggregates, potentially contributing to surface darkening and a reduction of glacial albedo.^6^

A suite of analytical methods was applied to investigate cryoconite particles from a single, ice-lidded cryoconite hole on the Canada Glacier in the McMurdo Dry Valleys, Antarctica, and elucidate the role of biofilms on glacial surfaces. A diverse microbial community associated with these particles was determined from 454 pyrosequencing to be dominated by *Cyanobacteria* (27%), *Actinobacteria* (24%), *Proteobacteria* (22%) *and Bacteroidetes* (19%; Supplementary Table S1). The photoautotrophic cyanobacteria were further identified by epifluorescent microscopy to be *Oscillatoria*, the dominant cyanobacterial genus present in cryoconite holes globally.^8^ To further explore interactions between auto- and heterotrophic organisms, *Bacteroidetes* were selected as they have been shown to be a dominant heterotrophic lineage (87%) within Antarctic cryoconite particles.^9^ Phylum-specific fluorescent *in situ* hybridisation probes targeting *Bacteroidetes* enabled the visualisation of cells within the *Oscillatoria* phycosphere biofilm (Supplementary Figure S3). Particle–cell interactions were visualised with confocal laser scanning microscopy using auto-fluorescence and nucleic acid specific fluorescent stains while simultaneously imaging the surface structure of individual cryoconite mineral particles (Figure 1). Unlike traditionally used techniques such as scanning electron microscopy (SEM), this novel approach allowed for both the visualisation and quantification of hydrated cellular and biofilm biomass. Imaging hydrated biofilms exposes them to a constant fluid shear throughout the image collection process, reaffirming the presence of biofilm structure and the attachment of cells to a surface, rather than artificial attachment as a result of fixation or dehydration processes commonly used in SEM. Importantly, there was no non-specific staining of combusted cryoconite mineral particles for any stain used in this study (Figure 1f), confirming that all fluorescently stained material was biotic in nature. Beyond visualisation of particle–cell interactions, total cellular biomass represented 14.5±1.26% (*n*=10 images) of the cryoconite sediment surface area, while autofluorescent, cellular biomass covered 1.70±0.861% (*n*=10 images). Calcofluor White, a stain that binds to cellulose and polysaccharides^10^ was used to identify the presence of EPS in the biofilm. EPS-like material covered 19.2±1.02% (*n*=11 images) of the total surface area and was imaged as diffuse non-microbial staining, unassociated with cellular-like structures, which is another clear indication of the presence of biofilm. Combined, ~35% of the cryoconite sediment surface was covered by biofilm.

To further elucidate the role of biofilm in glacial surface microbial community interactions a nanoscale secondary ion mass spectrometry^11^ (nanoSIMS; Supplementary Information) approach was applied in combination with halogenated *in situ* hybridisation^12^ using ^13^C- bicarbonate and ^15^N- ammonium for labelling experiments. All filamentous *Oscillatoria* cells analysed (*n*=37) were significantly enriched in ^13^C and ^15^N above background compared to the natural isotope abundance of cryoconite (^13^C atom %=1.074 and ^15^N atom %=0.366). Incorporation of ^13^C-labelled bicarbonate and ^15^N ammonium ranged between 1.2–3.0 fmol C per cell per h and 0.10–0.35 fmol N per cell per h, respectively. Incorporation of newly released cyanobacterial ^13^C-exudates and ^15^N by heterotrophic *Bacteroidetes* cells (*n*=137) decreased with increasing distance to *Oscillatoria* biofilms, with cells close to cyanobacteria being significantly more enriched in ^13^C (two-way analysis of variance (ANOVA), F=45.2, *P*<0.0001) and ^15^N (two-way ANOVA, F=89.4, *P*<0.0001; Figure 2c). These results indicate that biofilm on cryoconite sediments provides a matrix for cell arrangements, increases nutrient and energy transfer between community members and allows heterotrophs in close proximity to autotrophs to effectively scavenge excreted products; factors critical for survival and proliferation in these extreme environments. Such close cellular interactions are of particular importance in Antarctic cryoconite as they may remain entombed over annual to decadal scales, promoting microbial processes that recycle resources.^13^

Cryoconite particles were composed of primary minerals such as silicate oxides, cordierite and orthoclase. Calcite was identified as an associated secondary mineral-weathering product (Supplementary Figure S2). Total organic matter accounted for 7.7% of the cryoconite dry weight (Supplementary Table S3), which was higher than previously reported for Canada Glacier,^9^ but bracketed the lower range for Arctic cryoconite.^6^ To further investigate the quality and composition of OC in the cryoconite environment we utilised excitation emission spectroscopy (Supplementary Figure S1) and thermogravimetric analyses. The OC associated with the cryoconite particles was composed of 88.5% labile OM, dominated by carbohydrates (Supplementary Table S2). These compositional characteristics suggest a microbial origin of the OM and resemble the types of compounds derived from microbial exudation processes.

Biofilms have been found in diverse environments and proven to be ecologically advantageous for survival.^14^ Our unprecedented data show evidence of prominent biofilm formation on Antarctic cryoconite mineral particles, where the close arrangement of heterotrophs and autotrophs promotes increases in cellular activity enabling a highly efficient nutrient transfer between community members. It has been estimated that ~4.5% or 365,184 m^2^ of the Canada Glacier is covered by cryoconite.^9^ Taken together with results from our study this suggests that ~127,814 m^2^ of the Canada glacier surface could potentially be covered by biotic material. Considering the average number of photosynthetic days (226),^15^ the amount of cryoconite sediment on the surface of the Canada Glacier,^9^ and the experimentally determined cell-specific rate of carbon fixation, we estimated that *Oscillatoria* cells may fix 1.60 kg C within cryoconite across the Canada Glacier per season. A recent study,^16^ which was synchronised with the sample collection in this study, showed that cryoconite hole communities exhibited net autotrophy with an estimated total carbon fixation potential of 9.07 kg C per season across the surface of the Canada Glacier. As such, *Oscillatoria* cells may contribute ~20% of the total seasonal C fixation. Bacterial productivity in glacial environments is strongly influenced by the quality and quantity of fixed OC;^13^ thus, it is important to consider both bulk and species-specific primary production. However, we acknowledge that these extrapolations are greatly oversimplified with biological processes inferred from selected community members (i.e. *Bacteroidetes* and *Oscillatoria*) hosted within a single cryoconite hole.

Antarctic cryoconite have been shown to be ‘hotspots’ of biological activity; herein we show that cryoconite biofilm may have a significant role in promoting this activity.^9^ Further, as the transport and deposition of black carbon is less in Antarctica than in the Arctic,^17^ our data indicate that microbial processes may have a substantial effect on the accumulation of OM on cryoconite particles; pertinent findings considering that OM covering Antarctic cryoconite particles has been shown to reduce the reflectance of visible light.^7^ Thus, we hypothesise that future increases in temperature and longer melt seasons will stimulate biofilm communities and the accumulation of organic matter on cryoconite particles. The importance of biofilm formation in cryoconite holes merits further research to determine their development, the formation of larger aggregates composed of organic and inorganic material, and thus, their potential for reducing surface albedo of glaciers.

## Figures and Tables

**Figure 1:**
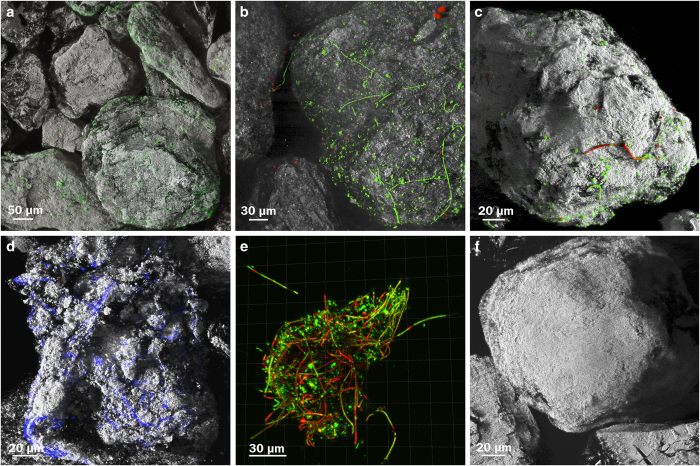
Representative confocal laser scanning microscopy images of cryoconite sediment with associated microbial communities and biofilm. (**a**) Green=SYBR Green stained microbes, grey=reflection of the sediment. (**b**,**c**) Red=auto-fluorescencing cells, green=SYBR Green stained microbes, grey=reflection of the sediment. (**d**) Blue=Calcofluor White stained EPS, grey=reflection of the sediment. (**e**) Red=Propidium Iodide (membrane compromised cells) Green=Syto9 stain (live cells). (**f**) Control image of combusted cryoconite sediment following described staining protocol, grey=reflection of the sediment.

**Figure 2:**
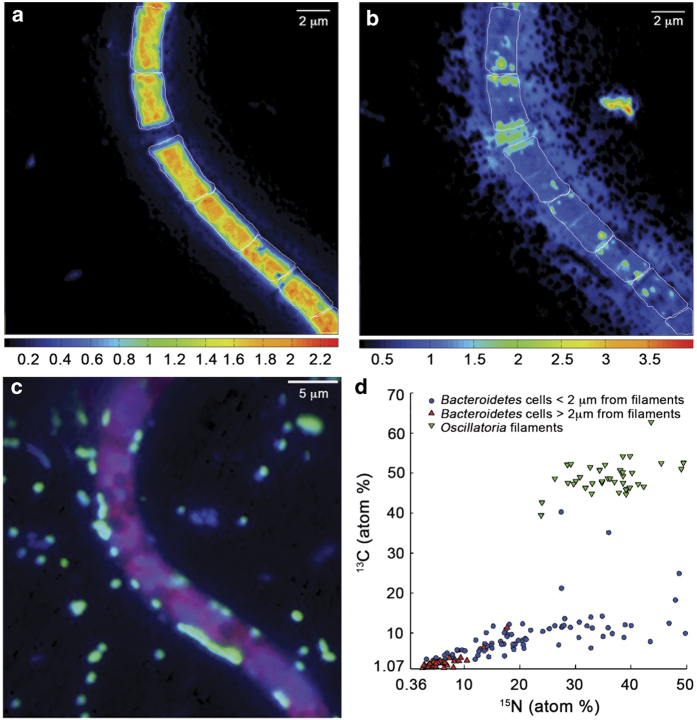
Example of nanoSIMS isotope ratio images of an analysed *Oscillatoria* sp. filament for **a** the ^13^C/^12^C ratio (**b**) the ^15^N/^14^N ratio (**c**) the epifluorescence overlay used to confirm cell identification of hybridised *Bacteroidetes* cells (green), DAPI stained (blue) and an autofluorescent filament (red). White lines indicate regions of interest (ROIs) section of an analysed *Oscillatoria* sp. filament. (**d**) NanoSIMS analysis of ^13^C and ^15^N enrichment measurements atom % (AT%) for *Bacteroidetes* sp. cells based on proximity to filamentous *Oscillatoria,* and *Oscillatoria* cells (▼). Cells <2 μm from a filamentous cell (●), and cells >2 μm from a filamentous cell (▲).
