# Supplementary material for: Biofilms on glacial surfaces: hotspots for biological activity
Source: NPJ Biofilms Microbiomes. 2016 Jun 8;2:16008–. doi: 10.1038/npjbiofilms.2016.8 (PMC5515272; doi:10.1038/npjbiofilms.2016.8)
Supplement: Supplementary Information [file npjbiofilms20168-s1.pdf]

Biofilms on glacial surfaces: hotspots for biological activity

H. J. Smith[1,2], A. Schmidt[1,3], R. Foster[4], S. Littmann[5], M. M. M. Kuypers[5], and C. Foreman[1,3]**

Supporting Information:

<u>Supplementary Information Content:</u>

Supplementary Materials and Methods

Supplementary Results

Supplementary References

Supplementary Figures (S1-S3)

Supplementary Tables (S1-S3)

Supplementary Materials and Methods:

Study Site and Sample Collection and Preparation

The cryoconite hole used in this study was collected during the 2011 Austral summer from the surface of the Canada Glacier (77°37’ S, 162°57’ E) as part of a larger sampling campaign (n=32) [1]. The ice lidded cryoconite hole was 38 cm by 34 cm with a maximum depth of 25 cm. The water depth of the sampled cryoconite hole was 12 cm and the depth of sediment was 0.5 cm. Liquid water and sediment were extracted from the glacial surface using syringes and ethanol cleaned spatulas and placed into sterile Whirl-Pak® bags. All samples were stored for three weeks at -20°C prior to further processing. Sediments (117.14 g of sediment in total) were prepared for different analytical methods in two ways; (i) 2 g were placed into sterile cryo vials and stored at -80°C for confocal laser scanning microscopy (CLSM), and (ii) 40 g were added to 500 ml of DI water and placed on an orbital shaker at 1,500 rpm at 10°C for 24 hrs as required for single cell secondary ion mass spectrometry (nanoSIMS) analyses. After shaking, the supernatant containing the dislodged bacterial cells were separated from the sediment and immediately used for subsequent analyses (see below nanoSIMS preparation section). Cryoconite from the Canada Glacier have been studied over the last several years and there is information available regarding cryoconite distribution, biogeochemical data, community structure, and exposure intervals to atmospheric conditions for cryoconites from this location [2-7]. Relevant for this study is the previously published distribution of cryoconite holes across the Canada Glacier [2] as well as rates of primary production and isolation age [1].Therefore, a single sample from the well-studied glacial surface was advantageous for the methodologically intensive nature of this study.

Bacterial Community Composition

Genomic DNA was extracted from 0.25 g of cryoconite sediment using a MO BIO PowerSoil™ DNA Isolation Kit, following the manufacturer's protocol (MO BIO Laboratories Inc., Carlsbad, CA, USA). The extracted DNA was cleaned and concentrated with the Wizard® SV Gel and PCR Clean-Up System (Promega Corporation, Madison, WI, USA) following established manufacturers' protocols. Small subunit rRNA gene sequences were amplified with barcoded universal bacterial primers FD1 (5′-ctcgcgtgtcAGAGTTTGATCCTGGCT- CAG-3′) and 529R (5′-ctcgcgtgtcCGCGGCTGCTGGCAC- 3′) spanning the V1, V2 and V3 regions. The amplification protocol began with a hot start (94°C for 4 min), followed by 28 cycles of a 30 s 94°C denaturation, a 1 min annealing step at 58°C, and an elongation step at 72°C for 1 min. After the completion of the 28 amplification cycles, there was a final elongation at 72°C for 10 min. PCR products were excised from a 0.8 % agarose gel and cleaned using an Ultrafree®-DA gel extraction column (Millipore Corporation, Bedford, MA, USA). The gel extract was cleaned and concentrated after the gel extraction using the Wizard® SC Gel and PCR Clean-Up System and the dsDNA PCR product was quantified with a Qubit fluorometer (Invitrogen, Carlsbad, CA, USA). Adaptor sequences were ligated onto the amplicon products and sequencing was performed using a 454 GS Jr. (454 Life Sciences, Branford, CT, USA). Sequences were trimmed to one standard deviation below the mean sequence length, and the data was processed using the Quantitative Insights Into Microbial Ecology (QIIME) toolkit [8]. Briefly, sequences were clustered into OTUs using a 97% identity threshold, and the most abundant sequence

from each OTU was selected as a representative sequence for the OTU. Taxonomy was assigned for each representative sequence using the Basic Local Alignment Search Tool (BLAST) against the Silva database. Chimeric sequences were removed with Chimera Slayer [9] and chimeric sequences were confirmed using Bellerophon [10]. Sequence data were deposited in the NCBI SRA database under the accession number SRP073064.

NanoSIMS Sample preparation

As surface charging strongly influences secondary ion formation on particle surfaces, incubations of cryoconite microbes were conducted using the detached cells in the supernatant. Samples were incubated with ^{15}N-labeled ammonium chloride (~99% ^{15}N, Cambridge Isotopes Laboratories Inc., Andover, MA, USA, 0.1mM final concentration) and ^{13}C-labeled sodium bicarbonate (~99% ^{13}C, Cambridge Isotopes Laboratories Inc., Andover, MA, USA, 1mM final concentration) in 1 L bottles in a light incubator to simulate *in situ* light (50.6 photons per m^2) and temperature (4°C) conditions for 72 hrs. After incubation samples were fixed with paraformaldehyde (final concentration 2%) for 1.5 hrs at room temperature. Subsamples (10 ml) were then filtered onto gold-palladium coated 0.2 μm GTTP polycarbonate filters (Millipore) under low vacuum pressure (< 7 psi) and washed three times with 1X phosphate buffered saline (PBS). Filters were dried and stored in cryovials at -20°C until further processing.

Hybridization and microscopic evaluation

Sections (5 mm diameter) of the gold-paladium coated filters containing cells were

excised and hybridized with Fluorescent *In Situ* Hybridization (FISH) with horseradish peroxidase (HRP)-labeled oligonucleotide probes following the Halogen *In Situ* Hybridization Seconday Ion Mass Spectrometry (HISH-SIMS) protocol described by [11]. To avoid detachment of cells through sample preparation protocols the 5 mm spheres were initially embedded in 0.1% low melting point agarose. For the analysis of gram–negative *Bacteroidetes* bacteria, cells were permeabilized with a lysozyme (10 mg ml^{-1} in 0.05 M EDTA (pH 8.0) and 0.1M Tris-HCl) treatment at 37°C for 1 hr. After permeabilzation filters were washed three times with ultrapure water (MQ, Millipore) and endogenous peroxidases were bleached with 3% H_2O_2 for 10 min at room temperature. Prior to hybridization filters were washed three times for 1 min with ultrapure water and submerged in 96% ethanol for 1 min and allowed to air dry completely. Filters were subjected to a 1 hr pre-hybridization step without the probe, then probes were added (1:150 v/v) and hybridized at 46°C for 6 hrs using a previously described protocol[11]. The following oligonucleotide probes were used to probe *Bacteroidetes* lineages: CF319a (Probe Sequence (5'-3'): TGG TCC GTG TCT CAG TAC, 16S target position: 319-336, formamide concentration: 35%) and CF319b (Probe Sequence (5'-3'): TGG TCC GTA TCT CAG TAC, 16S target position: 319-336, formamide concentration: 35%) [12]. Hybridized cells were counterstained with 4',6'-diamindino-2-phenylindole (DAPI) at a final concentration of 1 µg mL^{-1} in the dark for 10 min. The 5 mm filter sections were covered in mountant (4 parts Citifluor (Citifluor , Ltd., London, United Kingdom), 1 part VectaShield (Vector Laboratoires, Burlingame, CA)) and 10 randomly chosen fields (grid size of 15625 μm^2) corresponding to 1,000-1,200 DAPI stained cells were counted using a Zeiss Axioskop II fluorescence microscope (Zeiss, Berlin, Germany) with a final

magnification of 1,000X. Counts are reported as mean averages from triplicate cell counts of cells g^{-1} of sediment that have been converted based on phylogenetic classification to percentages of the total number of cells counted. *Bacteroidetes* sp. cells identified with FISH and *Oscillatoria* sp. cells of interest were identified by morphology and excitation under blue (450-490 nm) and green (510-560 nm) excitation filter sets and imaged. Areas of interest for nanoSIMS were marked using a Laser Micro Dissection (LMD) microscope 6500 (Leica, Wetzlar, Germany). Microscopic pictures were taken and used for orientation purposes during the subsequent nanoSIMS analysis and for post processing using look@nanoSIMS software (see below).

NanoSIMS analysis

NanoSIMS analysis was performed using a Cameca NanoSIMS 50 L instrument (Cameca, Gennevilliers, France) at the Max Planck Institute for Marine Microbiology in Bremen. After re-identifying the area of interest marked by the LMD with the nanoSIMS CCD camera, samples were pre-sputtered with a Cesium (Cs^+) primary ion beam with a current of ~300 pA. For the measurements a primary ion beam with a beam current between 1 and 3 pA and a beam size <100 nm was used to raster the analyzed area. The measurements were performed with an image size of 256 x 256 pixels over a chosen raster size of 20 x 20 µm with a dwelling time of 1 or 2 ms per pixel. Mass resolving power in the majority of the measurements was >8000. Negative secondary ion images of $^{12}C^-$, $^{13}C^-$, $^{19}F^-$, $^{12}C^{14}N^-$, $^{12}C^{15}N^-$ and $^{32}S^-$ were recorded simultaneously with the multi-collection system of the instrument. All scans (40-50 planes) were corrected for drift of the beam and sample stage after acquisition and accumulated using the "Look@NanoSIMS" software package (Polercky et al. 2012). Region of interest (ROIs)

were defined using the $^{19}F^{-}$ images representing the HISH labeled *Bacteroidetes* sp. cells, and ROIs for the non-labeled filamentous cyanobacterial cells were chosen based on the $^{12}C^{14}N$ image compared to fluorescent images for DAPI stained cells.

The equations used for ^{13}C and ^{15}N assimilation were as previously described in[13]. The cell diameter for both cyanobacterial cells and *Bacteroidetes* sp. were estimated from nanoSIMS images after cells were circled using the Look@NanoSIMS software analysis package [14]. Cell heights for both cell types were determined using a Nanoscope III extended-multimode atomic force microscope (Vecco, Santa Barbara, CA). Underestimations of cell diameter are possible due to the fixation step (2% PFA for 1.5 hrs). The cell diameter measures were used in equations for bio-volume estimation as described in Sun and Lui (2003)[15] For filamentous cyanobacterial cells, the bio volume was estimated using the equation of a cylinder:

$$V = (\pi/4) \text{ x } d^2 \text{ x } h) \qquad (1)$$

where d is cell diameter and h is cell height. Average height for individual cyanobacterial filaments was equal to 1.57 µm.

For estimating bio volume of individual *Bacteroidetes* sp. cells the equation of a sphere was used

$$(V = (\pi/6) \text{ x } d^3) \qquad (2)$$

where d is cell diameter. The initial cellular carbon content was estimated based on the relationship between bio volume and carbon content previously described in Verity at al. (1992) [16]:

$$Log[C] = -0.363 + (0.863 \text{ x } (Log(V)) \quad (3)$$

Logarithms (LOG) are base 10 and C content was used to estimate the N content. As none of the analyzed organisms are isolated, standard elemental analysis was not possible and therefore we based our estimates of initial C and N content on the Redfield ratio (C:N) of 6.6.

Cell specific C (A_C)and N (A_N) assimilation was calculated following the equations presented in Foster et al. (2013) and are as follows:

$$A_C = (^{13}C_{ex} \text{ x } C_{inital})/C_{SR} \quad (4)$$

$$A_N = (^{15}N_{ex} \text{ x } N_{inital})/N_{SR} \quad (5)$$

where $^{13}C_{ex}$ and $^{15}N_{ex}$ is the atom (AT) % of ^{13}C and ^{15}N, respectively, corrected for by the mean value of the respective ratios in non-enriched bulk samples (time 0) measured by elemental analysis isotope ratio mass spectrometry (EA-IRMS) analyses. The $C_{initial}$ and $N_{initial}$ variables are the initial C and N content as presented in equation 3, while C_{SR} and N_{SR} are the labeling percentage of ^{13}C and ^{15}N, respectively, in the experiment. The calculated assimilation values were divided by the incubation time (h) to determine the cell specific C fixation and N fixation rates. It should be noted that there is the potential for a dilution of ^{13}C and ^{15}N signals as a result of the HISH-SIMS fixation procedures, and hence the calculated uptake rates potentially underestimate rates of incorporation [17].

It was recently highlighted that microbial cells interact at the microscale [18], to investigate these microscale interactions and to quantify the C and N transfer from

autotrophic *Oscillatoria* sp. to heterotrophic *Bacteroidetes* sp., the enrichment (AT% 13C and AT% 15N) measured in the *Bacteroidetes* cells was divided into two groups and include values from cell residing: close (< 2 µm) or far (> 2 µm) from a *Oscillatoria* sp. cell. Phytoplankton exudates are known to form diffusion limited chemical gradients around the cell surface, [19,20] the diffusivity of this boundary layer can be estimated using the relationship between cell size and organic matter leakage calculated according to Jackson (1987) [20]. Where ϕ is the relationship between the cell radius and cell carbon content, as determined by Mullin et al. (1966) [21] for planktonic photoautotrophic organisms:

$$\phi = ba^{2.28} \qquad (1)$$

Where *b* is a constant equal to 2.67 x 10^{-4} mol multiplied by the cells radius *a* = 1.99 $\mu m^{2.28}$. Organic matter leakage was subsequently calculated according to Jackson (1987) [20]:

$$C = \left(\frac{L}{4\pi D}\right) r^{-1} + C_{\infty} \qquad (2)$$

Where the carbon leakage rate of L = *f*µϕ. Standard values from Jackson (1987) were used for the following parameters: where *f* is the organic matter leakage rate from photoautotrophic cells, µ is the specific growth rate, *D* is the molecular diffusivity of organic matter, r^{-1} is the distance from the cell surface. C_{∞}, the concentration in the bulk which was assumed to be nominally small and therefore set to zero. The concentration of

exuded organic matter was then calculated incrementally for distances ranging from very near the cell surface (0.1 μm) to 10 μm away from the cell surface. To preserve these distances and inhibit cell loss or movement through sample preparation and analysis filters were covered with a thin layer of low met agarose (0.1%) after the initial sample filtration. Using the *stats* package in R [22] a two-way ANOVA with section and distance as factors was used to separately analyze ^{13}C and ^{15}N AT% enrichment of both the close and far *Bacteroidetes* sp. cells. The model included either ^{13}C AT% or ^{15}N AT% as a response to an interaction effect added for each analyzed section and distance from filament.

CLSM imaging and data processing

For imaging, cryoconite particles were thawed on ice, placed into a sterile petri dish and stained for 20 min in the dark. Fully hydrated cryoconite particles were stained with either SYBR Green, a nucleic acid stain (Invitrogen; Life Technologies) 40X final concentration; or calcofluor white, which is typically used for staining of chitin, cellulose and polysaccharides [23] (Fluka; Sigma-Aldrich) final concentration 1 μM. After staining, cryoconite particles were rinsed with 0.2 μm filter sterilized DI water three times to remove any excess stain. Stains were chosen for this study after comparing a variety of different stains to determine which stains would be the most sample appropriate and the least reactive with abiotic material. Sediment samples were imaged with a Leica TCS SP5 II upright confocal microscope using either a 25X water immersion objective, 0.95 NA, WD 2.5 mm; or a 63X water immersion objective, 0.9 NA, WD 2.2 mm. Fluorophore excitation lasers and emission bandwidths are as follows:

SYBR Green (ex 497/em 520) 488 nm excitation, 500–550 nm emission collection; autofluorescence, 561 nm excitation, 580–700 nm emission collection; calcofluor white (ex 355, em 433), 405 nm excitation, 450–490 nm emission collection; and reflection imaging, 488 nm excitation. A minimum of 10 randomly selected images were collected to enumerate cellular biomass (SYBR green, autofluoresence, reflection) and biotic material biomass (calcofluor white and reflection). Z-stacks were collected in either 0.54 µm steps for the 25X objective or 0.64 µm steps for the 63X objective. To ensure that the stains selected for this study were not binding to abiotic material cryoconite particles were combusted at 450°C for 5 hrs to remove any biotic material present. Combusted particles were then stained and imaged following the same protocol as previously described. The 3D structure of individual cryoconite particles and associated biotic material was reconstructed from the CLSM images using IMARIS software (version 7.6.4). Surface area (μm^2) for each channel imaged was determined using the surface area calculation function in the IMARIS software. Surface area calculation parameters were optimized to sample and image specific properties. For the autofluorescent and SYBR Green images used to quantify individual cells images were smoothed at a surface area detail level of 0.5 μm^2, thresholding was based on a background subtraction method, and all voxels greater than zero were collected. Biofilm images were processed with the same parameters described above, with the exception that all voxels greater than 10 were collected. Sediment surface area was calculated the same way as for biofilm except that the surface was divided in half based on the inability of light to penetrate the sediment samples and the resulting overestimation of surface area. Calculated surface areas for cells identified with SYBR Green or autofluoresence, and biotic material were all

normalized to the surface area of cryoconite particles in the corresponding field of view analyzed.

Fluorescence Spectroscopy

Dissolved organic matter (DOM) extracted from cryoconite sediment was analyzed using excitation emission spectroscopy (EEMs). Scans were collected over an excitation range of 240–450 nm in 10 nm increments and emission was monitored from 300–560 nm in 2 nm increments on a Fluoromax-4 spectrofluorometer (HORIBA Jobin-Yvon). Samples were analyzed for UV absorbance with a Thermo Scientific Genesys 10 scanning UV spectrophotometer from 190-1100 nm on optically dilute samples (absorbance values < 0.3 at 254 nm). EEMs data were post-processed to correct for instrument-specific bias using manufacturer-generated correction files for excitation, emission, and blank subtraction. Specific regions of fluorescence were defined for each carbon source corresponding to previously identified natural organic matter fluorophores [24]. Fluorescence intensity values in the proteinaceous regions (B and T fluorophores) were summed and classified as more-labile, while humic-like fluorescence regions (A and C fluorophores) were combined and classified as less-labile and more recalcitrant. The fluorescence for both defined regions was individually summed for the two samples (cryoconite water and sediment extracted DOM).

Sediment Analysis

Powder X-ray diffraction (XRD) was used to determine the mineralogical composition of studied cryoconite sediment. Samples were ground into a homogenous powder with a

mortar and pestle to homogenize the sediment mixture. XRD analysis was completed with a Scintag XGen-4000 X-ray Powder Diffraction Spectrometer. X-ray spectra were generated across a 2θ range of 5-70° in 0.02° steps with a scan rate of 2° min^{-1} for an initial survey and across regions of prominent peaks at 24 0.5° min^{-1} for greater detail. X-ray spectra were analyzed using the Scintag Diffraction Management System for NT (DMSNT) computer software package to determine peak positions and relative intensities and subsequently compared to the International Centre for Diffraction Data (ICDD) powder diffraction file catalog for identification of crystalline minerals. Stepwise thermo-gravimetric analysis was conducted to determine bulk organic matter content and composition following methods from [25]. Briefly, 500 mg sub-samples of cryoconite sediment (in triplicate for each combustion temperature) were weighed and placed into crucibles and dried for three days at 38ºC. Individual samples were weighed to obtain sample dry weight. Samples were placed in a muffle furnace at four different combustion temperatures (105ºC, 200ºC, 350ºC, and 520ºC) for 4 hrs. After combustion samples were placed into a desiccator to cool, once cool samples were weighed for the second time. The difference in the dry weight and the weight post combustion for each sample represents the percent organic matter lost at different temperatures of ignition. These percent differences were used to calculate the total organic content and different lability classifications which has been previously described by [25-27]. The following differentiations were used: the loss of water (38-200°C), thermolabile (200-350°C), and stable organic matter (350-520°C) [28].

Chemical Analyses

Samples run in triplicate for chlorophyll a (chl *a*) extraction and analysis were filtered in the dark through 25 mm pre-combusted GF/F filters wrapped in aluminum foil and kept at -20°C until. Chl *a* was extracted in a 1:1 solution of 90% and dimethyl sulfoxide for 12 hrs in the dark at -20°C. Extracted chl *a* was analyzed with a Turner 10-AU fluorometer. Anion samples in triplicate were filtered through 0.4 μm 47 mm nucleopore filters, deionized water was used as a filtration blank, and samples were analyzed on a Dionex ICS-1100 ion chromatography system.

Supplementary Results:

Fluorescent DOM was detected in both the cryoconite water and in the sediment extracted DOM. The fluorescent signature of the sediment extracted DOM has fluorescence in the B and T regions, compared to the water where fluorescence was only present in the T regions. Similarly between both samples fluorescence was not detected in the humic-like regions, rather the fluorescent DOM present was predominately in the more-labile, microbially derived regions. The sediment extracted DOM was comprised of 89.07% more-labile DOM and 10.92% less labile DOM, and the overlaying water consisted of 93.57% more-labile and 6.42% less labile DOM.

Simulation of organic matter leakage from a cell with a relatively large leakage rate (0.5 d^{-1}[20]) under standard conditions showed that there was an inversely proportional relationship between the concentration of exuded organic matter and the distance from the filament surface. Within the phycosphere of a filamentous cell, with a 1.99 μm radius, 10% of the cell surface concentration was estimated to extend 1um away of the cell

surface, and at 2 μm from the cell surface only 5% of the initial concentration will extend (Figure SI 3). We therefore conclude that >2 μm is a conservative delineation where exuded organic matter concentrations are too diffuse and below the threshold for bacterial chemosensory detection.

Supplementary References

1. Telling, J. *et al.* Spring thaw ionic pulses boost nutrient availability and microbial growth in entombed Antarctic Dry Valley cryoconite holes. *Front Microbiol* **5,** 694–694 (2014).
2. Fountain, A. G., Tranter, M., Nylen, T. H., Lewis, K. J. & Mueller, D. R. Evolution of cryoconite holes and their contribution to meltwater runoff from glaciers in the McMurdo Dry Valleys, Antarctica. *Journal of Glaciology* **50,** 35–45 (2004).
3. Fountain, A. G., Nylen, T. H., Tranter, M. & Bagshaw, E. Temporal variations in physical and chemical features of cryoconite holes on Canada Glacier, McMurdo Dry Valleys, Antarctica. *J. Geophys. Res.* **113,** G01S92 (2008).
4. Foreman, C. M., Sattler, B., Mikucki, J. A., Porazinska, D. L. & Priscu, J. C. Metabolic activity and diversity of cryoconites in the Taylor Valley, Antarctica. *J. Geophys. Res.* **112,** G04S32 (2007).
5. Bagshaw, E. A., Tranter, M. & Wadham, J. L. High-resolution monitoring reveals dissolved oxygen dynamics in an Antarctic cryoconite hole. *Hydrol. Process.* **25,** 2868–2877 (2011).
6. Bagshaw, E. A. *et al.* Biogeochemical evolution of cryoconite holes on Canada Glacier, Taylor Valley, Antarctica. *J. Geophys. Res.* **112,** (2007).
7. Bagshaw, E. A. *et al.* Do Cryoconite Holes have the Potential to be Significant Sources of C, N, and P to Downstream Depauperate Ecosystems of Taylor Valley, Antarctica? *Arctic, Antarctic, and Alpine Research* **45,** 440–454 (2013).
8. Caporaso, J. G. *et al.* QIIME allows analysis of high-throughput community sequencing data. *Nat Meth* **7,** 335–336 (2010).
9. Haas, B. J. *et al.* Chimeric 16S rRNA sequence formation and detection in Sanger and 454-pyrosequenced PCR amplicons. *Genome Res.* **21,** 494–504 (2011).
10. Huber, T., Faulkner, G. & Hugenholtz, P. Bellerophon: a program to detect chimeric sequences in multiple sequence alignments. *Bioinformatics* **20,** 2317–2319 (2004).
11. Musat, N. *et al.* A single-cell view on the ecophysiology of anaerobic phototrophic bacteria. *Proc. Natl. Acad. Sci. U.S.A.* **105,** 17861–17866 (2008).
12. Manz, W., Amann, R., Ludwig, W., Vancanneyt, M. & Schleifer, K.-H. Application of a suite of 16S rRNA-specific oligonucleotide probes designed to investigate bacteria of the phylum cytophaga-flavobacter-bacteroides in the natural environment. *Microbiology* **142**, 1097-1106 (1996).
13. Foster, R. A. *et al.* Nitrogen fixation and transfer in open ocean diatom–cyanobacterial symbioses. *ISME J* **5,** 1484–1493 (2011).
14. Polerecky, L. *et al.* Look@NanoSIMS--a tool for the analysis of nanoSIMS data in environmental microbiology. *Environ. Microbiol.* **14,** 1009–1023 (2012).
15. Sun, J. Geometric models for calculating cell biovolume and surface area for

phytoplankton. *Journal of Plankton Research* **25,** 1331–1346 (2003).
16. Verity, P. G. *et al.* Relationships between cell volume and the carbon and nitrogen content of marine photosynthetic nanoplankton. *Limnol. Oceanogr.* **37,** 1434–1446 (1992).
17. Musat, N. *et al.* The effect of FISH and CARD-FISH on the isotopic composition of ^{13}C- and ^{15}N-labeled Pseudomonas putida cells measured by nanoSIMS. *Systematic and Applied Microbiology* 1–10 (2014).
18. Stocker, R. The 100 μm length scale in the microbial ocean. *Aquat Microb Ecol* **76,** 189–194 (2015).
19. Bell, W. & Mitchell, R. Chemotactic and Growth Responses of Marine Bacteria to Algal Extracellular Products. *Biological Bulletin* **143,** 265 (1972).
20. Jackson, G. A. Simulating chemosensory responses of marine microorganisms. *Limnol. Oceanogr.* **32,** 1253–1266 (1987).
21. Mullin, M. M., Sloan, P. R. & Eppley, R. W. Relationship Between Carbon Content, Cell Volume, and Area in Phytoplankton. *Limnol. Oceanogr.* **11,** 307–311 (1966).
22. R Development Core Team. R: a language and environment for statistical computing. Vienna, Austria: R Foundation for Statistical Computing. (2012).
23. Chen, M.-Y., Lee, D.-J., Tay, J.-H. & Show, K.-Y. Staining of extracellular polymeric substances and cells in bioaggregates. *Appl Microbiol Biotechnol* **75,** 467–474 (2007).
24. Coble, P. G. Characterization of marine and terrestrial DOM in seawater using excitation-emission matrix spectroscopy. **51,** 325–346 (1996).
25. Kristensen, E. Characterization of biogenic organic matter by stepwise thermogravimetry (STG). *Biogeochemistry* **9,** 135–159 (1990).
26. Walter E Dean, Jr. Determination of Carbonate and Organic Matter in Calcareous Sediments and Sedimentary Rocks by Loss on Ignition: Comparison With Other Methods. *SEPM JSR* **Vol. 44,** 242–248 (1974).
27. Langford, H., Hodson, A., Banwart, S. & Bøggild, C. The microstructure and biogeochemistry of Arctic cryoconite granules. *Annals of Glaciology* **51,** 87–94 (2010).
28. Cuypers, C. *et al.* Amorphous and condensed organic matter domains: the effect of persulfate oxidation on the composition of soil/sediment organic matter. *Chemosphere* **48,** 919–931 (2002).

Supplementary Figures:

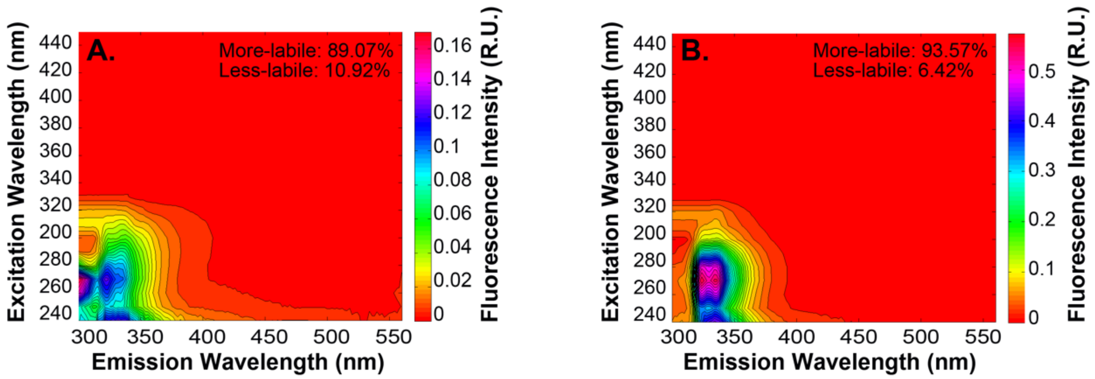

Figure S1: 3D EEM of cryoconite DOM extracted from sediment (A.) and cryoconite water (B.) and corresponding percentages of more- and less-labile components.

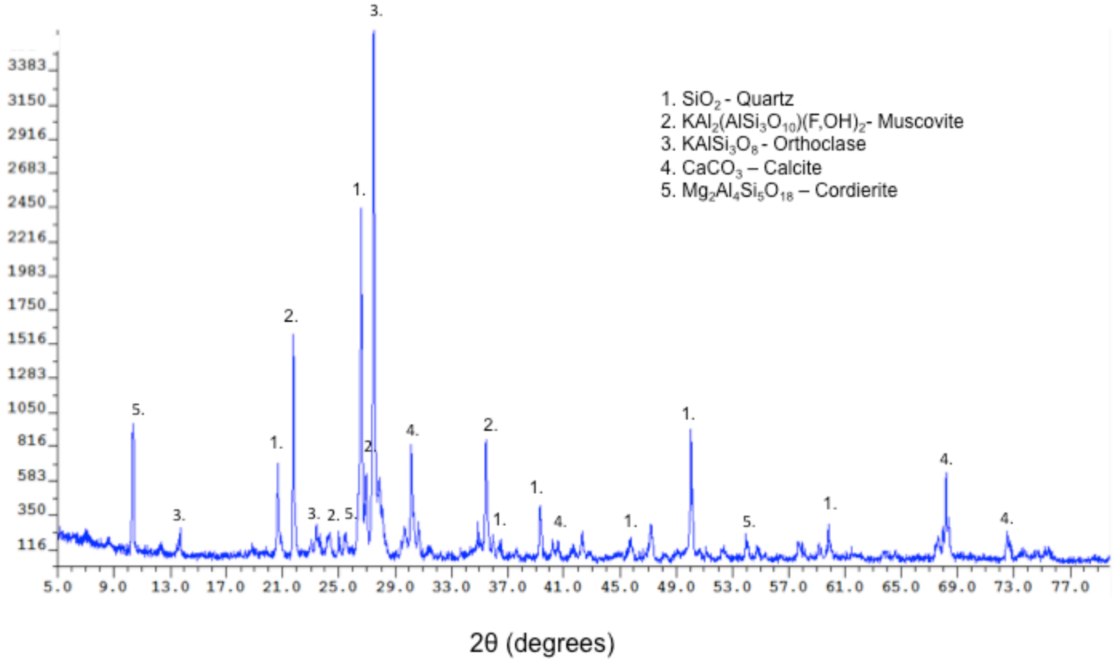

Figure S2: Powder XRD spectral profile of cryoconite sediments, used to illustrate the interpretation of present constituents. Cryoconite sediment diffraction patterns suggest a dominance of silicate materials, specifically quartz and corresponding weathering products.

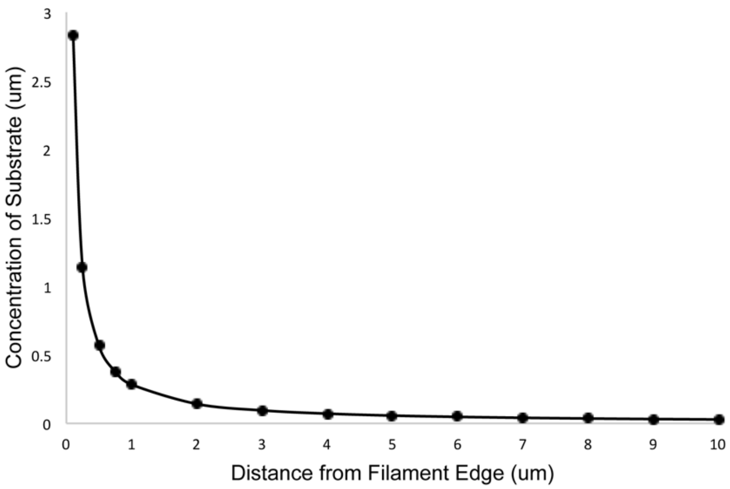

Figure S3: Concentration of exuded organic matter in the boundary layer of a cell with a 1.99 μm radius, assuming an exudation rate of 0.5 d^{-1} (Jackson 1987).

Supplementary Tables:

**A. Bacterial Community**		**B. Cyanobacterial Community**	
Phylum Level Taxonomic Classification	Relative Abundance (%)	Genus Level Taxonomic Classification	Relative Abundance (%)
Acidobacteria	0.94	*Tolypothrix*	8.61
Actinobacteria	23.69	*Chamaesiphon*	6.55
Bacteroidetes	18.81	*Pseudanabaena*	5.84
Chloroflexi	0.04	*Nostoc*	12.16
Cyanobacteria	27.37	*Phormidium*	4.56
Firmicutes	3.80	*Oscillatoria*	61.30
Gemmatimonadetes	1.93	Unclassified	0.98
Planctomycetes	1.46		
Proteobacteria	21.68		
Unclassified	0.29		

Table S1: Comparison of the relative abundances of 454 Pyrosequencing sequences for A.) dominant cryoconite sediment organisms at the phylum taxonomic classification level and B.) Cyanobacterial sequences at the genus classification.

Temperature	Sample Average % Dry Weight ± SD	Classification	Description
38-105°C	0.49 ± 0.73	Loss of Water	Crystalline lattice water, and hygroscopic water of salts and organic matter
105-200°C	4.3 ± 2.08		
200-350°C	2.19 ± 1.02	Thermolabile	Dominated by carbohydrates
350-520°C	0.76 ± 0.80	Stable Organic Matter	Oxidation of aromatic groups (lignin, humic substances, kerogens) and char

Table S2: Triplicate thermo-gravimetric analysis of organic matter (as percentage of dry weight) from cryoconite sediments, and characterization of organic matter present (Kristensen, 1990). Total organic matter accounted for 7.7% of the cryoconite dry weight.

OM (% of dry weight)	Cl^- (mg/L)	NO_2^- (mg/L)	NO_3^- (mg/L)	Br^- (mg/L)	PO_4^{-3} (mg/L)	SO_4^{-2} (mg/L)	Chl a (μg L^{-1})	Bacteria (cells/g)
7.7	7.31	0.17	0.19	0.1956	0.1998	6.1846	0.22	1.86 x 10^{8}(±1.49 x 10^{6})

Table S3: Biogeochemistry of cryoconite water and bacterial cell abundances plus or minus the standard deviation (OM= organic matter, Chl a = chlorophyll a)
